# Cerebral Amyloid Angiopathy With Progressive Cortical Superficial Siderosis and Convexity Subarachnoid Hemorrhage in an Apolipoprotein E (APOE) ε2/ε2 Homozygous Patient: A Case Report and Literature Review

**DOI:** 10.7759/cureus.87007

**Published:** 2025-06-29

**Authors:** Masahiro Hayashi, Mayumi Ikeda, Katsuji Kobayashi

**Affiliations:** 1 Psychiatry and Neurology, Medical Corporation Asanokawa Sakuragaoka Hospital, Kanazawa, JPN; 2 Psychiatry and Neurobiology, National Hospital Organization Hokuriku Hospital, Nanto, JPN; 3 Psychiatry, Awazu Neuropsychiatric Sanatorium, Komatsu, JPN

**Keywords:** apoe ε2 allele, cerebral amyloid angiopathy, convexity subarachnoid hemorrhage, cortical superficial siderosis, gait disturbance, hemorrhagic lesion, intracerebral hemorrhage, white matter lesion

## Abstract

Sporadic cerebral amyloid angiopathy (CAA) is characterized by progressive amyloid β-protein (Aβ) deposition in small cortical and leptomeningeal vessels. It is associated with intracerebral hemorrhage (ICH), cognitive decline, and gait disturbance. Among its hemorrhagic manifestations, cortical superficial siderosis (cSS) and convexity subarachnoid hemorrhage (cSAH) are gaining increasing attention, particularly in individuals carrying the apolipoprotein E (APOE) ε2 allele. A cSAH typically represents an acute-phase lesion caused by blood leakage into the subarachnoid space, often presenting as a single linear hypointensity on MRI. In contrast, cSS is a chronic-phase lesion characterized by hemosiderin deposition in the superficial cortex, typically appearing as a double-line hypointensity. Both lesions are believed to originate from the rupture or leakage of structurally fragile small vessels in the leptomeninges and superficial cortex. The accumulation of iron-containing blood products may trigger inflammatory responses and secondary damage to vessels and brain parenchyma, promoting recurrent hemorrhage and cognitive decline. The APOE ε2 allele is hypothesized to exacerbate vascular fragility through Aβ-mediated mechanisms and to increase the severity of hemorrhagic complications in CAA. Homozygosity for APOE ε2 is rare in the general population and has been associated with an elevated risk of hemorrhage in CAA, making this case particularly noteworthy.

We report the case of a probable CAA in a man in his 60s who was homozygous for APOE ε2. Over a 10-month period, he experienced progression from mild cognitive impairment and subtle gait abnormality to severe dementia and marked limping. Imaging revealed recurrent cSAH and progressive cSS, along with simultaneous ICH and cSAH in opposite hemispheres. In particular, atypical cSAH-characterized by larger volume or prolonged blood retention-was followed by more extensive cSS in the same regions. Brain perfusion single-photon emission computed tomography (SPECT) showed hypoperfusion in cortical areas affected by these lesions, particularly in the bilateral frontal lobes, more pronounced on the left, and in the bilateral parietal lobes. This case highlights the aggressive course of CAA in a patient with APOE ε2/ε2 homozygote, where repeated hemorrhagic events and iron accumulation may result in both vascular injury and parenchymal dysfunction. We discuss imaging, clinical progression, and pathophysiological implications, with a focus on the role of APOE ε2 in exacerbating hemorrhagic lesions and promoting cognitive and motor decline.

## Introduction

Sporadic cerebral amyloid angiopathy (CAA) is characterized by the progressive deposition of amyloid β-protein (Aβ) in the walls of small blood vessels within the cerebral cortex and leptomeninges [1]. While traditionally recognized as a major cause of intracerebral hemorrhage (ICH) in the elderly, CAA has also been increasingly associated with cognitive decline [2].

Among its hemorrhagic manifestations, cortical superficial siderosis (cSS) and convexity subarachnoid hemorrhage (cSAH) have emerged as key neuroimaging markers of CAA [3]. In CAA cases presenting with cSAH, cSS is frequently observed, and cSAH is now considered a potential acute-phase manifestation of cSS [4,5]. Reflecting this relationship, the 2022 revision of the Boston Criteria (Version 2.0) incorporated cSAH as a diagnostic imaging feature across all CAA categories [5].

On axial T2-weighted or susceptibility-weighted imaging (SWI), MRI sequences that are sensitive to blood products, the cSAH typically appears as a linear hypointensity reflecting acute blood accumulation in the subarachnoid space. Whereas cSS represents a chronic-phase lesion characterized by hemosiderin deposition in the superficial cortex, often appearing as a double-line hypointensity. Both cSS and cSAH have been implicated not only in the onset and recurrence of ICH but also in their progression and the development of cognitive impairment.

These hemorrhagic manifestations are also associated with the apolipoprotein E (APOE) ε2 allele. Although both the ε2 and ε4 alleles are linked to increased CAA severity and hemorrhagic risk [4,6,7], their mechanisms of vascular injury differ. In ε4 carriers, vascular damage primarily stems from Aβ accumulation within vessel walls, whereas in ε2 carriers, it is related to structural fragility of small vessels in the leptomeninges and superficial cortex, rendering them more susceptible to rupture [7,8].

These ε2-related changes are believed to underlie the development of cSS and cSAH [3,4,7]. Histopathologically, cSS is marked by hemosiderin deposition in the superficial cortex, accompanied by vascular and parenchymal injury mediated by iron toxicity and inflammatory responses [2,9]. Here, we report a rare case of CAA in a man in his 60s who was homozygous for the APOE ε2 allele, a genotype found in less than 2% of the general population [10].

The patient presented with progressive cognitive decline and gait disturbance, along with marked progression of hemorrhagic lesions, including expansion of cSS and cSAH, and simultaneous occurrence of cSAH and ICH in opposite hemispheres. Notably, cSAH lesions often evolved from atypical forms, characterized by prolonged persistence and/or deviation from the typical single-line appearance. In this report, we explore the pathophysiological mechanisms underlying cSS and cSAH and examine their associations with ICH, cognitive decline, and the APOE ε2 genotype.

## Case presentation

A 64-year-old right-handed man with a long-standing independent lifestyle and over 30 years of employment as a construction worker presented with progressive cognitive decline and gait disturbance. Prior to presentation, he had no known vascular risk factors such as hypertension, diabetes mellitus, or use of antithrombotic medications, and no significant comorbidities, history of head trauma, or relevant family history of cerebrovascular disease were identified. Four months prior to his initial visit, he experienced a transient loss of consciousness. Head CT performed at the time showed no abnormalities. Two months later, he began forgetting the steps of familiar tasks. By the fourth month, he was making frequent errors and taking longer to complete simple tasks, prompting a referral to our neurology department.

At presentation, his blood pressure was 128/82 mmHg, and blood tests were unremarkable. Neurocognitive evaluation revealed preserved orientation and recent memory, but impaired backward digit span and calculation. His mini-mental state examination (MMSE) score was 24, and the Montreal cognitive assessment (Japanese version) score was 20, consistent with dysfunction in executive function, attention, and calculation. Neurological examination was normal, but his timed up and go (TUG) test took 12 seconds (cutoff: 10 seconds) [11], with mild external rotation of the right lower limb and reduced gait speed.

**Figure 1:**
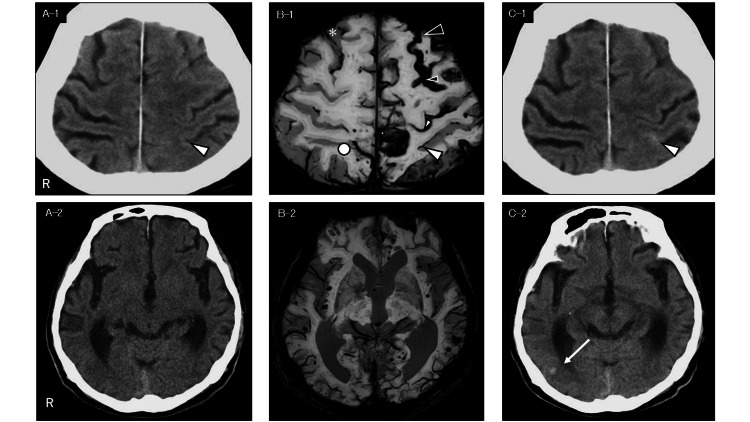
Imaging from the initial visit and the seventh month post presentation A-1, A-2: Initial CT revealed diffuse cerebral atrophy; B-1: The SWI shows acute cSAH in the left superior frontal sulcus and a thin, discontinuous hypointense line in the left postcentral sulcus. A previously unrecognized cSAH was also identified in the right parietal lobe (white circle); B-2: Bilateral CMBs were noted on SWI; C-1, C-2: The CT at stroke onset showed hyperdense lesions in the left postcentral sulcus with ill-defined margins and an ovoid hyperdensity in the right occipital lobe White arrowhead: Left postcentral sulcus; Black arrowhead: Anterior portion of the left superior frontal sulcus; Small black arrowhead: Posterior portion of the left superior frontal sulcus; Small white arrowhead: Left central sulcus; White asterisk: Anterior portion of the right superior frontal sulcus; White arrow: Hemorrhagic foci in the right occipital lobe; Symbols are consistent across A–1 to C–2. SWI: Susceptibility-weighted imaging; cSAH: Convexity subarachnoid hemorrhage; CMB: Cerebral microbleed

**Figure 2:**
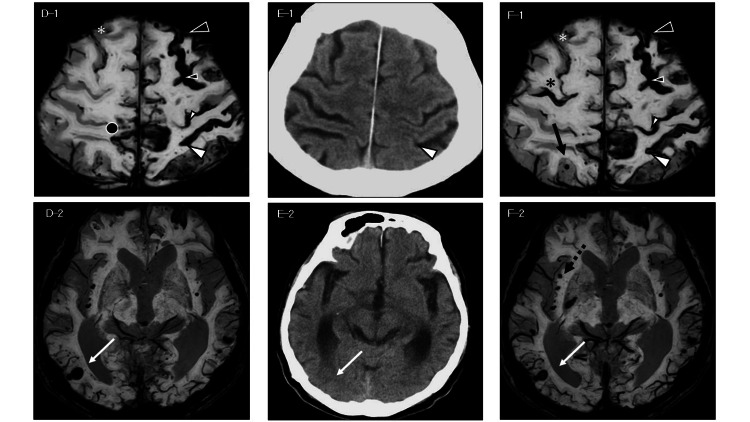
Follow-up imaging from seven to 10 months in the patient with probable CAA with APOE ε2/ε2 D-1: The SWI two days later reveals a single-line hypointense signal in the left postcentral sulcus (white arrowhead) suggesting acute cSAH. In addition, a new cSAH is observed in the right parietal lobe, adjacent to a pre-existing cSAH (black circle); D-2: A new ICH is noted in the right occipital lobe (white arrow) on SWI; E-1, E-2: Follow-up CT showed resolution of the previously identified hyperdense lesions; F-1: The SWI at 10 months demonstrates a distinct double-line hypointense signal in the left postcentral sulcus (white arrowhead), consistent with cSS. A new cSAH is seen in the right frontal lobe (black asterisk), along with a new CMB in the right parietal lobe (black arrow); F-2: An additional CMB appears near the external capsule in the right hemisphere (black dotted arrow) Black arrowhead: Anterior portion of the left superior frontal sulcus; Small black arrowhead: Posterior portion of the left superior frontal sulcus; White arrowhead: Left postcentral sulcus; Small white arrowhead: Left central sulcus; White asterisk: Anterior portion of the right superior frontal sulcus; White arrow: Hemorrhagic foci in the right occipital lobe. Symbols are consistent across D–1 to F–2. SWI: Susceptibility-weighted imaging; ICH: Intracerebral hemorrhage; cSAH: Convexity subarachnoid hemorrhage; cSS: Cortical superficial siderosis; CMB: Cerebral microbleed; CAA: Cerebral amyloid angiopathy; APOE: Apolipoprotein E

Initial CT revealed diffuse cerebral atrophy (Figure 1 A-1 and A-2). Although further evaluation had been planned, the patient was lost to follow-up. He returned four months later following an episode of disorganized speech and inability to use household appliances. At admission, he was alert and communicative but had poor recollection of the preceding day, suggestive of a transient confusional state. The MMSE score had declined to 17, with marked deficits in registration and orientation. Gait speed had worsened, and the TUG score increased to 13 seconds.

Subsequent follow-up over 10 months revealed progressive hemorrhagic changes, including recurrent cSAH, new cerebral microbleeds (CMBs), and cSS development (Figure 2). Key clinical and imaging findings over the 10-month follow-up are summarized in Table 1.

**Table 1:** Summary of main clinical events, symptoms, functional assessments, and corresponding imaging panels (Figures 1 and 2) over a 10-month period in our patient with probable CAA (APOE ε2/ε2 genotype) ADL: Activities of daily living; CMBs: Cerebral microbleeds; cSAH: Convexity subarachnoid hemorrhage; cSS: Cortical superficial siderosis; ICH: Intracerebral hemorrhage; MMSE: Mini-mental state examination; SWI: Susceptibility-weighted imaging; TUG: Timed up and go test; CAA: Cerebral amyloid angiopathy; APOE: Apolipoprotein E

Figure reference	Month of follow-up visit	Main clinical event	Main clinical symptoms	MMSE (/30)	TUG (sec)	Imaging findings
Figure 1: Panels A-1 and A-2	0	Initial visit	Mild cognitive decline, slight gait abnormality	24	12	Diffuse cerebral atrophy
Figure 1: Panels B-1 and B-2	4	First admission	Post-confusional state (alert on admission)	17	13	Acute cSAH in left superior frontal sulcus; ccattered cSS (left hemisphere)
Figure 1: Panels C-1 and C-2	7	Second admission	Transient symptoms at stroke onset (e.g., headache, dizziness)	Not reported	Not reported	Bilateral hyperdensities
Figure 2: Panels D-1 and D-2	7.1	Follow-up (during hospitalization)	No stroke symptoms	Not reported	Not reported	Left acute cSAH; right acute ICH; new right parietal cSAH
Figure 2: Panels E-1 and E-2	7.5	Follow-up (during hospitalization)	Basic ADL intact	12	14	Resolution of bilateral hyperdensities
Figure 2: Panels F-1 and F-2	10	Follow-up (routine outpatient visit)	Severe dementia; limping gait	8	16	Conversion of left cSAH to cSS; right frontal cSAH expansion; new CMBs

**Figure 3:**
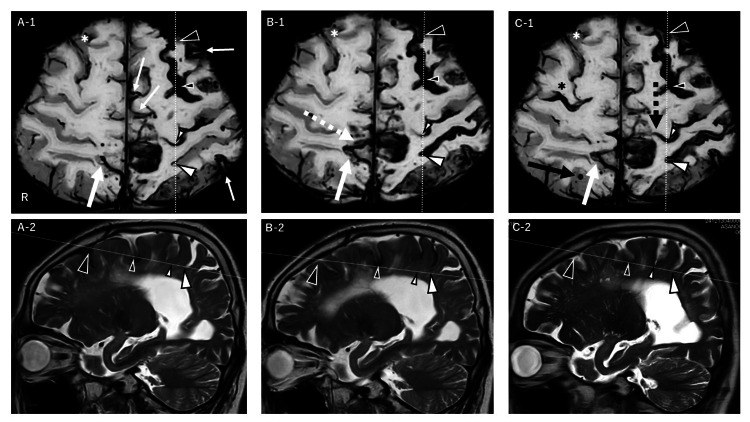
Serial axial SWI and sagittal T2WI MRI showing CSAH and CSS progression A-1, A-2: Four months post-initial visit, SWI shows linear hypointensities consistent with cSAH in left frontal sulcus. A hypointense line is noted in right frontal lobe, with discontinuous or double-line signals in right superior frontal sulcus. The left postcentral sulcus shows a thin hypointensity; left hemisphere demonstrates typical cSS (small white arrows). The T2-sagittal images show marked low signal in the anterior left frontal sulcus, milder posteriorly. The central sulcus (small white arrowhead) has hypointensity in the sulcal space and superficial cortex; the postcentral sulcus is hyperintense. B-1, B-2: Seven months after initial visit, the anterior left superior frontal sulcus evolves into blurred double-line hypointensity; posterior changes are minimal. New cSAH appears in left postcentral sulcus and right parietal lobe (white dotted arrow). The T2WI shows reduced anterior sulcal signal. The central sulcus appears narrowed with faint residual hypointensity; low-signal core emerges in postcentral sulcus. C-1, C-2: 10 months after initial visit, SWI shows a clear double-line in anterior left sulcus; posterior remains stable. New cSAH is seen in right superior frontal sulcus. The central sulcus shows medial hypointense extension (black dotted arrow); left postcentral sulcus shows cSS. The T2WI shows anterior low signals and resolved; posterior ones persist. The central sulcus widens with hyperintensity and subtle residual low signal. Black arrowhead: Anterior left superior frontal sulcus; Small black arrowhead: Posterior segment; White arrowhead: Left postcentral sulcus; Small white arrowhead: Left central sulcus; White asterisk: Right superior frontal sulcus; White arrow: Hemorrhagic focus in the right parietal lobe; Vertical dotted line: Sagittal plane (A-2 to C-2); Oblique dotted line: Axial plane (A-1 to C-1) SWI: Susceptibility-weighted imaging; T2WI: T2-weighted imaging; cSAH: Convexity subarachnoid hemorrhage; cSS: Cortical superficial siderosis

Detailed imaging findings corresponding to these clinical events are described below. The SWI showed linear hypointensities along the left superior frontal sulcus and central sulcus, consistent with cSAH, along with additional hypointensities in the right frontal and parietal sulci. A thin, discontinuous hypointense line was seen in the left postcentral sulcus. In addition, scattered typical cSS lesions were noted in the left hemisphere. Multiple bilateral CMBs were also observed (Figure 1 (B-1and B-2) and Figure 3 A-1). Corresponding sagittal T2-weighted images (T2WI) revealed hypointensity in the left superior frontal sulcus, indicative of acute subarachnoid blood (Figure 3 A-2). Figure 3 illustrates the spatial correspondence between the SWI and T2WI findings, highlighting acute cSAH in the superior frontal sulcus and the distribution of typical cSS lesions.

Electroencephalography (EEG) revealed no epileptiform discharges but showed dominant slow alpha activity around 8 Hz. Cerebrospinal fluid (CSF) analysis revealed normal glucose and cell count, but elevated protein (67 mg/dL) and positive globulin. Total tau was within normal limits at 152 pg/mL (reference range: 146-410 pg/mL). The CSF Aβ biomarkers were not assessed; however, given the normal tau level and the presence of characteristic imaging findings, the absence of Aβ data did not preclude a diagnosis of CAA in this case. Genetic testing confirmed homozygosity for APOE ε2/ε2. Based on the clinical and imaging findings, the patient was diagnosed with probable CAA according to the Boston Criteria Version 2.0.

During hospitalization, the patient remained independent in basic activities of daily living (ADL) and was subsequently discharged to a care facility. Two months later (seven months after the initial visit), he presented with nocturnal urinary incontinence, headache, dizziness, and nausea. By the time of evaluation, these symptoms had resolved. He was alert and ambulatory. A CT six hours after symptom onset showed hyperdense areas in the left postcentral sulcus and right occipital lobe, consistent with acute hemorrhage (Figure 1 C-1 and C-2).

Two days later, the SWI revealed a clearly defined hypointensity in the left postcentral sulcus, consistent with cSAH. In the right occipital lobe, two partially overlapping round hypointensities with slightly different shapes were observed, consistent with ICH. In addition, multiple tiny hypointense spots were also observed in sulci adjacent to the occipital ICH, suggestive of associated microbleeds or secondary hemorrhagic changes. A new double-line hypointensity appeared in the anterior left superior frontal sulcus, and a new cSAH appeared adjacent to a pre-existing cSAH in the right parietal lobe (Figure 2 D-1 and D-2; Figure 3 B-1). On sagittal T2WI, the left postcentral sulcus showed a sharply demarcated hypointensity, while hypointensities in and around the anterior left superior frontal sulcus had diminished. However, marked superficial cortical hypointensities had newly emerged, delineating the cortical contours more clearly (Figure 3 B-2).

Repeat EEG showed no epileptiform activity but demonstrated dominant theta waves and diffuse slowing. A CT two weeks later confirmed near-complete resolution of the hyperdense lesions (Figure 2 E-1 and E-2). The MMSE score dropped to 12, and the TUG took 14 seconds. Despite imaging progression, the patient remained independent in basic ADL, including eating and toileting, and was returned to the care facility.

At 10 months after the initial visit, the patient exhibited further progression of cognitive and gait impairments. His MMSE score had declined to 8, and the TUG time increased to 16 seconds. Worsening right lower limb external rotation resulted in a limping gait. Additionally, marked apathy and diminished initiative were observed, with a corresponding decline in ADL.

**Figure 4:**
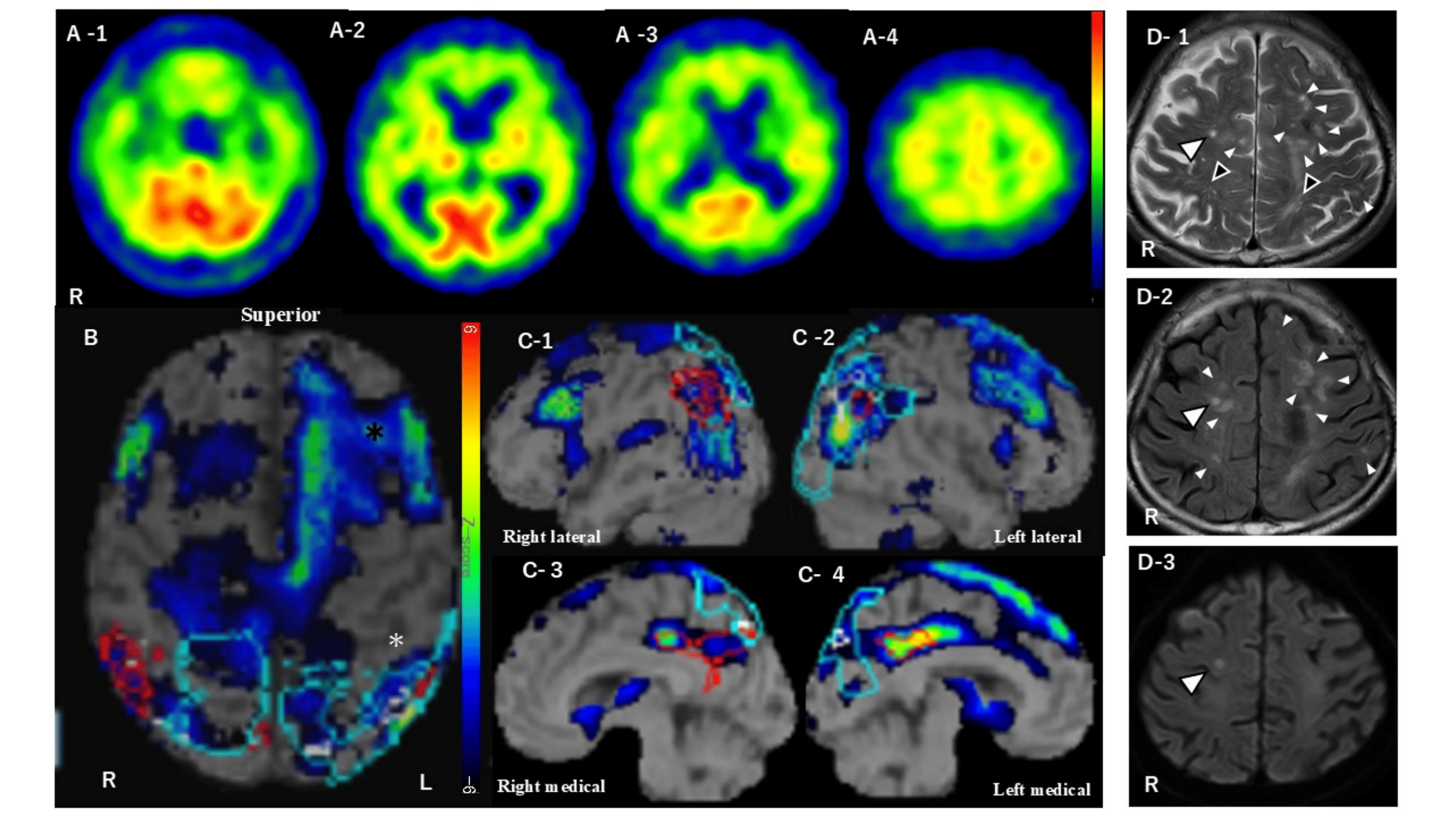
Cerebral blood perfusion SPECT and MRI findings 10 months after initial visit A-1 to A-4: Cerebral perfusion SPECT revealed a generalized reduction in cortical blood flow. In contrast, perfusion in the basal ganglia and thalamus was relatively preserved bilaterally. B, C-1 to C-4: The Z-score maps generated by the eZIS showed marked hypoperfusion in the left frontal lobe (black asterisk), with additional reductions in the right frontal lobe, bilateral parietal lobes, and the bilateral posterior cingulate gyrus. Notably, no hypoperfusion was observed in the left postcentral sulcus or adjacent regions (white asterisk). D-1, D-2: The T2WI (D-1) and FLAIR (D-2) axial MRI images. The CSO-PVS are visible on T2WI (black arrowheads). The WMH-MS are evident in both images, predominantly in the frontal lobes with left-sided dominance (white arrowheads and large white arrowhead). D-3: One hyperintense spot was detected among WMH-MS in the right frontal lobe on DWI (large arrowhead) SPECT: Single-photon emission computed tomography; eZIS: Easy Z-score imaging system; T2WI:T2-weighted imaging; FLAIR: Fluid-attenuated inversion recovery; DWI: Diffusion-weighted imaging; VPS-CSO: Visible perivascular spaces in the centrum semiovale; WMH-MS: White matter hyperintensities in a multispot pattern

Subsequent imaging studies demonstrated diffuse cortical hypoperfusion on cerebral perfusion SPECT using iodine-123 N-isopropyl-p-iodoamphetamine, with relative sparing of the basal ganglia and thalamus (Figure 4 A-1 to A-4). The easy Z-score imaging system (eZIS) analysis revealed marked hypoperfusion in the left frontal lobe, along with reduced perfusion in the right frontal, bilateral parietal, and bilateral posterior cingulate cortices. Interestingly, despite typical cSS on SWI, the left postcentral sulcus and adjacent postcentral gyrus showed no corresponding hypoperfusion (Figure 4 B and C-1 to C-4).

The MRI revealed centrum semiovale perivascular spaces (CSO-PVS) on T2WI (Figure 4 D-1) and white matter hyperintensities in a multispot pattern (WMH-MS) predominantly in the frontal lobes on T2WI and fluid-attenuated inversion recovery (FLAIR) (Figure 4 D-1 and D-2). One of the WMH-MS lesions in the right frontal lobe exhibited high signal intensity on diffusion-weighted imaging (DWI), suggestive of infarction (Figure 4 D-3).

Although the patient was normotensive during hospitalization, antihypertensive therapy was initiated after the occurrence of simultaneous cSAH and ICH, with a target systolic blood pressure below 120 mmHg and diastolic pressure below 80 mmHg. Despite this, follow-up imaging three months later demonstrated progression of hemorrhagic lesions. 

## Discussion

The cSS and cSAH findings observed in this case were not included in the original 1995 Boston Criteria (v1.0), which recognized ICH and CMBs as the primary imaging markers for CAA [12,13]. To improve diagnostic accuracy, subsequent studies incorporated histopathological validation and reassessed CAA-specific imaging features. The 2010 revision, Boston Criteria version 1.5 (v1.5), introduced cSS as a key imaging finding, reportedly observed in approximately 60% of CAA cases. This addition increased diagnostic sensitivity for possible and probable CAA from 89.5% (v1.0) to 94.7% (v1.5) [12].

Furthermore, cSAH, which is closely associated with cSS, is increasingly recognized as an acute-phase manifestation of the same pathological process. In the most recent Boston Criteria v2.0, published in 2022, cSAH was incorporated as a new hemorrhagic marker across all diagnostic categories, from definite to possible CAA [5]. This updated classification is summarized in Table 2.

**Table 2:** Boston criteria version 2.0 for sporadic cerebral amyloid angiopathy *Other causes of haemorrhagic lesion: Antecedent head trauma, haemorrhagic transformation of an ischemic stroke, arteriovenous malformation, haemorrhagic tumor, central nervous system vasculitis. CAA: Cerebral amyloid angiopathy, ICH: Intracerebral haemorrhage, TFNE: Transient focal neurologic episodes, CI: Cognitive impairment, CMB: Cerebral microbleed, cSS: Cortical superficial siderosis, cSAH: Convexity subarachnoid haemorrhage, CSO-PVS: Centrum semiovale perivascular spaces, WMH-MS: White matter hyperintensities in a multispot pattern The table was simplified without altering the original diagnostic content from 'The Boston criteria version 2.0 for cerebral amyloid angiopathy: a multicentre, retrospective, MRI-neuropathology diagnostic accuracy study' by Charidimou et al. (2022) [5].

Boston Criteria (Version 2.0)
1. Definite CAA
Full post-mortem examination demonstrating:
• Presentation with spontaneous ICH, TFNEs, cSAH, or CI/Dementia
• Severe CAA with vasculopathy
• Absence of other diagnostic lesion
2. Probable CAA with supporting pathology
Clinical data and pathologic tissue (evacuated hematoma or cortical biopsy) demonstrating:
• Presentation with spontaneous ICH, TFNEs, cSAH, or CI/Dementia
• Some degree of CAA in specimen
• Absence of other diagnostic lesion
3. Probable CAA
• Age ≥50 years
• Presentation with spontaneous ICH, TFNEs, or CI/Dementia
• ≥2 of the following strictly lobar haemorrhagic lesions on T2*-weighted MRI, in any combination: ICH, CMB, cSS/cSAH foci
OR
• 1 lobar haemorrhagic lesion + 1 white matter feature (Severe CSO-PVS or WMH-MS)
• Ⅰlobar haemorrhagic lesion +Ⅰ white matter feature (Severe CSO-PVS or WMH-MS)
• Absence of any deep haemorrhagic lesions (ICH, CMB) on T2*weighted-MRI
• Absence of other cause of haemorrhagic lesions*
• Haemorrhagic lesion in cerebellum not counted as either lobar or deep haemorrhagic lesion
4. Possible CAA
Clinical data and MRI demonstrating:
• Age ≥50 years
• Presentation with spontaneous ICH, TFNEs, or CI/Dementia
• Absence of other cause of haemorrhagic lesions*
• Haemorrhagic lesion in cerebellum not counted as either lobar or deep haemorrhagic lesion
• Ⅰstrictly lobar haemorrhagic lesion on T2*-weighted MRI: ICH, CMB, cSS/cSAH focus
OR
• Ⅰwhite matter feature (Severe CSO-PVS or WMH-MS)
• Absence of any deep haemorrhagic lesions (ICH, CMB) on T2*weighted-MRI
• Absence of other cause of haemorrhagic lesions*
• Haemorrhagic lesion in cerebellum not counted as either lobar or deep haemorrhagic lesion

The hemorrhagic mechanisms underlying cSS and cSAH, as well as their interrelationship, remain incompletely understood. However, both are believed to result from rupture of small vessels in the leptomeninges and superficial cortex. The cSAH is considered the acute-phase manifestation of such hemorrhage, whereas cSS represents the chronic sequela [3,5].

Histopathologically, cSS is characterized by hemosiderin-laden blood product accumulation in the superficial cortex. The iron-induced cytotoxicity, reactive astrocytosis, and inflammatory responses are thought to contribute to secondary injury of adjacent cortex and vessels, potentially leading to recurrent hemorrhage and cognitive decline [2,9,14]. In this case, comparison of axial SWI and sagittal T2WI of the left hemisphere suggested that cSAH may be classified into three types based on morphology and temporal evolution.

**Figure 5:**
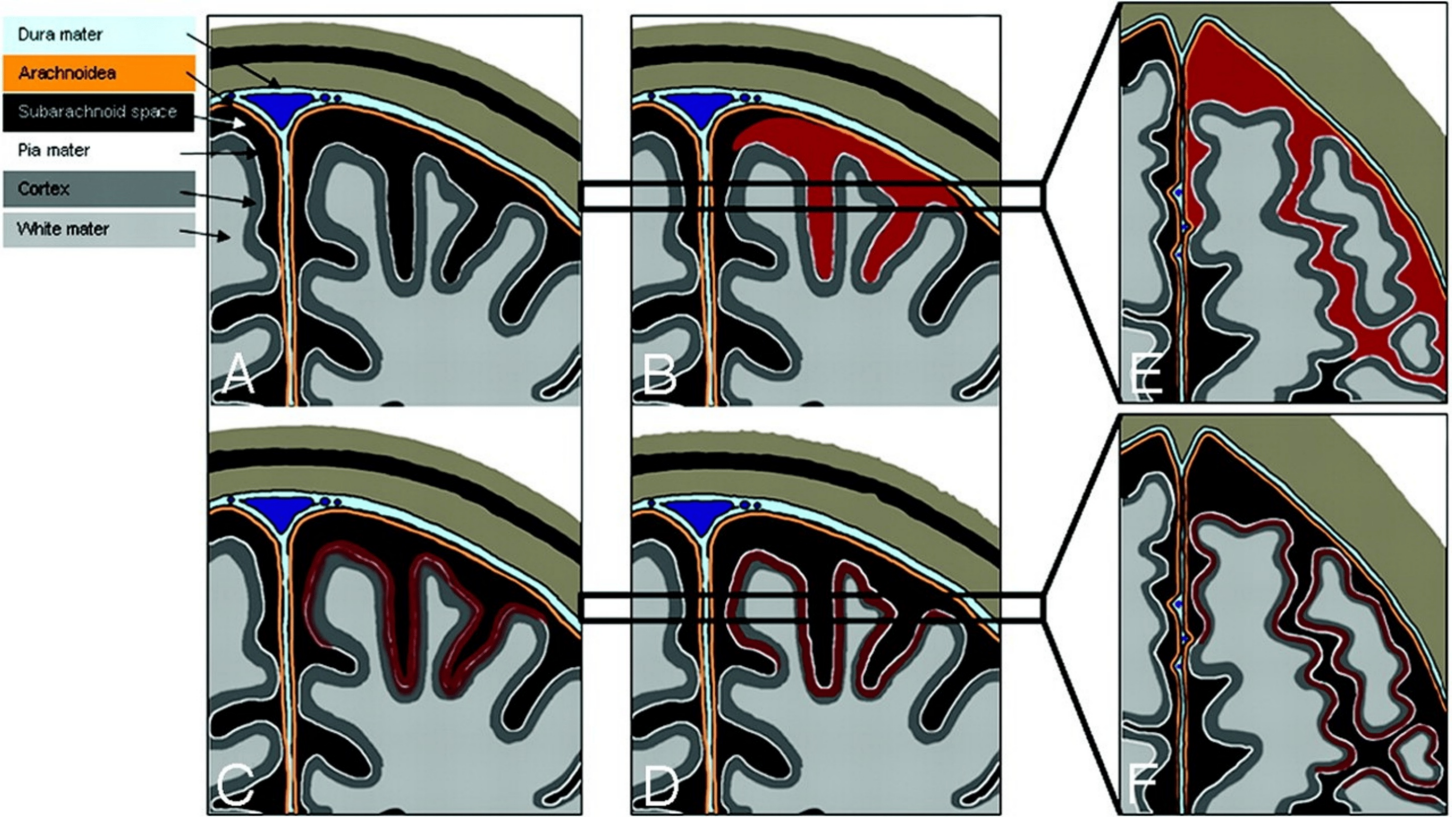
Schematic drawings illustrating subarachnoid hemosiderosis and superficial cortical hemosiderosis A-F: Schematic drawings illustrating subarachnoid hemosiderosis and superficial cortical hemosiderosis; A-D: Coronal schematic drawings, illustrating the time-dependent development of subarachnoid hemosiderosis and superficial cortical hemosiderosis (Black: Subarachnoid space; White line: Pia mater; Orange line: Arachnoid layer; Light blue: Dura mater); E and F: Horizontal schematic drawings corresponding to the areas marked with black bars in B and C, demonstrate the visual appearance of subarachnoid hemosiderosis and superficial cortical hemosiderosis on axial sections; A: Normal appearance of the subarachnoid space; B and E: Subarachnoid hemorrhage (red) presenting as a linear signal intensity in the subarachnoid space (E); C: Residues of blood penetrating the pia mater and deposit in the superficial layers of the cerebral cortex; D and F: Superficial cortical hemosiderosis, defined as linear residues of blood in the superficial layers of the cerebral cortex (dark red). Superficial cortical hemosiderosis typically has a bilinear “tracklike” appearance on axial sections, caused by the signal intensity of the normal-appearing subarachnoid space in the middle, which is bordered bilaterally by linear deposits of hemosiderin in the superficial layers of the adjacent cortex (F). Permission to reproduce the illustration from 'Acute convexity subarachnoid haemorrhage and cortical superficial siderosis in probable cerebral amyloid angiopathy without lobar haemorrhage' by Linn J et al. (2008) [3] was obtained through RightsLink.

These findings suggest that atypical cSAH may be associated with more severe parenchymal injury, contributing to regional hypoperfusion, white matter damage, and cognitive decline. The first type, observed in the left postcentral sulcus, corresponded to an acute hemorrhagic focus on CT. On SWI, it presented as a clearly defined single linear hypointensity. On sagittal T2WI, it appeared as a markedly hypointense area limited to the sulcus. Three months later, this resolved on T2WI, while SWI revealed a double-line hypointensity, consistent with cSS. This pattern is consistent with the pathological progression illustrated in Figure 5 [3].

The second type, found in the left superior frontal sulcus, initially appeared as a single-line hypointensity extending along the sulcus on SWI. Over time, cSS changes emerged only in the anterior portion, while cSAH persisted posteriorly. On T2WI, hypointensity in the subarachnoid space initially decreased and was replaced by superficial cortical hypointensity consistent with cSS. At six months, hypointensities persisted in the posterior portion of the left superior frontal sulcus, whereas the anterior portion showed signs of blood clearance and transition to cSS. This heterogeneous evolution implies that moderate-to-extensive subarachnoid hemorrhage may have caused delayed clearance, particularly in the posterior region.

The third type involved the left central sulcus, presenting with persistent single linear hypointensity for six months. The T2WI showed sulcal and superficial cortical hypointensity from the outset, indicating cSAH with early cSS. By month three, the sulcus narrowed and the hypointensity decreased, but by month six, it re-expanded with renewed hypointensity, suggesting recurrent hemorrhage. This re-expansion may have been triggered by a new hemorrhage in the adjacent postcentral sulcus. The SWI showed medial extension of the low-signal line, consistent with this hypothesis. Thus, this is considered a long-standing, atypical form of cSAH.

Although initially considered typical, the cSAH in the left postcentral sulcus was preceded by subtle, discontinuous hypointense signals on SWI three months earlier. This observation suggests that symptomatic cSAH may originate from pre-existing, asymptomatic lesions. Prior studies have reported new cSAH developing in regions with previous asymptomatic cSAH [4], implying the existence of atypical forms beyond currently recognized subtypes. These findings suggest that atypical cSAH, defined by prolonged retention of subarachnoid blood and subsequent iron deposition, may not only represent a transitional stage in the development of cSS, but also constitute a useful imaging biomarker indicative of heightened hemorrhagic susceptibility in CAA.

Another noteworthy feature of this case was the presence of two cerebral hemorrhages occurring simultaneously in different hemispheres, as detected by CT. In the absence of trauma, such findings are classified as primary multiple simultaneous intracerebral hemorrhages (MSICHs), defined as either concurrent hemorrhages or a second bleed occurring within 24 hours of the first. This phenomenon is rare, accounting for approximately 5.6% of all primary ICH cases [15].

A comprehensive review of 667 MSICH-related articles [15] published between 1950 and 2013 identified 105 cases that met strict diagnostic criteria, with cerebral small vessel disease and CAA being the most common etiologies. Most cases involved bilateral deep hemorrhages in the basal ganglia (35 cases) or thalamus (19 cases). Only one case presented with simultaneous lobar hemorrhages in both occipital lobes, and none exhibited the hemispheric distribution observed in our patient.

The underlying pathophysiology of MSICH is believed to involve widespread small vessel fragility and impaired dynamic cerebral autoregulation (dCA), with two proposed mechanisms: simultaneous rupture of fragile vessels or a secondary hemorrhage triggered by acute blood pressure elevation following an initial bleed. Given that CAA is a panencephalic vasculopathy characterized by diffuse Aβ-mediated vessel wall damage, these dual hemorrhages may reflect underlying vascular vulnerability unique to this condition [16].

Regarding APOE genotype, both the APOE ε2 and ε4 alleles are known to be strongly associated with cerebral amyloid angiopathy (CAA) [1,7]. The CAA cases carrying the APOE ε4 allele are typically classified as CAA type 1, characterized by amyloid-β (Aβ) deposition predominantly in cortical capillaries. In this subtype, disease severity is reported to correlate with the extent of Aβ accumulation within the vessel walls [7,8].

In contrast, CAA associated with the APOE ε2 allele is more frequently classified as CAA type 2, in which Aβ preferentially accumulates in small arterioles of the leptomeninges and superficial cortex. Notably, APOE ε2 is also implicated in structural abnormalities of the vessel wall, namely, vessel cracking, detachment, delamination of the outermost layer of the tunica media, and fibrinoid necrosis-which are believed to increase vascular fragility and the risk of hemorrhage [7,8].

These pathological changes may contribute to the development of cSS, particularly through bleeding, into the leptomeningeal and superficial cortical regions [7]. Furthermore, previous studies have shown that all documented CAA cases in individuals homozygous for APOE ε2 exhibited histopathological findings consistent with CAA type 2 [8]. Accordingly, the present case-homozygous for APOE ε2-is likely to represent CAA type 2, and the marked progression of cSS and cSAH observed in this patient may be explained by this specific genetic background and the associated vascular pathology.

The CAA-related dementia is thought to arise through three primary mechanisms: direct brain damage from ICH, coexisting Alzheimer’s disease (AD) pathology, and CAA-specific microvascular changes [2,17]. Notably, over 80% of AD cases exhibit CAA pathology, suggesting a shared amyloid β (Aβ)-related mechanism. However, cognitive impairment associated with CAA often resembles that of vascular dementia (VaD), with executive dysfunction and slowed information processing being more prominent than memory impairment [1,2]. In the present case, the clinical features were more consistent with VaD than with typical AD.

In addition, gait disturbance was observed from an early stage in this case. Previous studies have reported that gait speed is significantly reduced in patients with CAA compared to those with AD [18]. This impairment is thought to result from disruption of frontal-subcortical circuits, including the basal ganglia, as well as damage to frontal white matter. In this case, frontal cSS and cSAH may have contributed to the gait abnormalities.

The Boston Criteria version 2.0 introduced severe CSO-PVS and white WMH-MS as supportive imaging markers for CAA, both of which were observed in the present case [5]. The CSO-PVS is thought to represent β-amyloid accumulation within perivascular spaces, while WMH-MS is considered to reflect the structural injury to cortical penetrating arteries. In this case, frontal WMH-MS lesions were clearly visible on T2WI/FLAIR, and one lesion exhibited diffusion restriction on DWI, suggestive of infarction. These findings imply that WMH-MS may reflect irreversible parenchymal injury rather than transient ischemic changes [5,13].

Cerebral perfusion SPECT further demonstrated diffuse cortical hypoperfusion. Proposed mechanisms underlying hypoperfusion in cerebral amyloid angiopathy include luminal stenosis and impaired dCA [19]. Visual correlation between eZIS and SWI revealed marked perfusion deficits in regions affected by cSS and cSAH, particularly in the left frontal lobe-where atypical cSAH was present, and in the right hemisphere, where new cSAH emerged from a pre-existing atypical lesion. In contrast, the left postcentral sulcus, which showed typical cSS on SWI, exhibited no corresponding hypoperfusion. These observations suggest that atypical cSAH may lead to more severe parenchymal injury, contributing to regional hypoperfusion, WMH development, and subsequent cognitive decline.

No established treatment exists for CAA, but strict blood pressure control is essential for the prevention of ICH. Notably, even prehypertensive levels-defined as systolic blood pressure between 120 and 139 mmHg, are associated with increased ICH risk. Current guidelines recommend maintaining systolic pressure below 120 mmHg and diastolic pressure below 80 mmHg [20].

Beyond blood pressure control, comprehensive risk stratification is essential in managing patients with CAA, particularly when considering antithrombotic therapy-including both antiplatelet and anticoagulant agents-or invasive procedures such as brain biopsy. In this context, APOE genotyping may serve as a useful adjunct in evaluating individual hemorrhagic vulnerability and guiding clinical decision-making, given the elevated risk associated with such interventions. While this report describes a single case, it underscores the potential significance of atypical cSAH and APOE ε2 homozygosity as indicators of increased hemorrhagic vulnerability and disease progression in CAA. These findings warrant further investigation in larger patient cohorts to establish their diagnostic and prognostic value.

## Conclusions

In this report, we presented a case of a man in his 60s with probable CAA carrying the rare APOE ε2/ε2 genotype. The patient exhibited progressive hemorrhagic lesions, including cSS and cSAH, along with cognitive decline and gait disturbance. In APOE ε2-associated CAA, hemorrhagic events such as cSS and cSAH are thought to result from rupture of structurally fragile small vessels in the leptomeninges and superficial cortex. Subsequent iron deposition may lead to secondary vascular and parenchymal injury, thereby increasing the risk of recurrent hemorrhage and associated cognitive impairment. Notably, atypical forms of cSAH, characterized by either larger volumes or prolonged retention of blood products, may result in higher local iron concentrations. These deposits can exert direct neurotoxic effects and trigger more robust iron-mediated inflammatory responses, potentially causing greater damage to small vessels and surrounding brain tissue.
